# Enhanced Anti-Obesity Activities of Red Mold Dioscorea When Fermented Using Deep Ocean Water as the Culture Water

**DOI:** 10.3390/md11103902

**Published:** 2013-10-15

**Authors:** Li-Chun Wang, Tzu-Ying Lung, Yi-Hsin Kung, Jyh-Jye Wang, Tsung-Yu Tsai, Bai-Luh Wei, Tzu-Ming Pan, Chun-Lin Lee

**Affiliations:** 1 Continuing Education School, National Taitung Junior College, Taitung 95045, Taiwan; E-Mail: egg681128@hotmail.com; 2 Department of Food Science, Fu Jen Catholic University, New Taipei 24205, Taiwan; E-Mail: tytsai@mail.fju.edu.tw; 3 Department of Life Science, National Taitung University, 684, Sec. 1, Chunghua Rd., Taitung 95092, Taiwan; E-Mails: tzu-ying10@srdc.org.tw (T.-Y.L.); yihsin0933@sunway.cc (Y.-H.K.); blwei@nttu.edu.tw (B.-L.W.); 4 Stone & Resource Industry R & D Center, Hualien 97356, Taiwan; 5 R & D Division, Sunway Biotechnology Company Limited, Taipei 11494, Taiwan; 6 Department of Nutrition and Health Science, Fooyin University, Kaohsiung 83102, Taiwan; E-Mail: FT054@fy.edu.tw; 7 Department of Biochemical Science and Technology, National Taiwan University, Taipei 10617, Taiwan; E-Mail: tmpan@ntu.edu.tw

**Keywords:** deep ocean water, obesity, *Monascus*, monascin, ankaflavin

## Abstract

Deep ocean water (DOW) has, in previous studies, been found to be a novel anti-obesity drink and useful in raising *Monascus*-produced monascin and ankaflavin levels. This may resolve the limited anti-obesity ability of red mold dioscorea (RMD) known as the *Monascus purpureus*-fermented *Disocorea batatas*. This study aims to compare the anti-obesity effect of DOW-cultured RMD (DOW-RMD) and ultra-pure water-cultured RMD (UPW-RMD) in rats fed on a high fat diet. Moreover, the effect of ions composition of DOW and DOW-influenced functional metabolites change of RMD on the differentiation and lipogenesis regulation were investigated using 3T3-L1 pre-adipocytes. In the animal test, compared to UPW-RMD, DOW-RMD possessed better ability to inhibit increases in weight gain, and better feed efficiency, body-fat pad and cross-sectional area of adipocytes. In the cell test, the anti-obesity abilities of DOW-RMD in inhibiting PPARγ and C/EBPα expression in differentiation and lipoprotein lipase activity in lipogenesis were contributed to by the DOW-increased monascin and ankaflavin levels and the ions of DOW, respectively.

## 1. Introduction

Obesity is associated with a higher risk of developing diabetes and cardiovascular disease. At the cellular level, enlargement of the adipose tissue mass has been characterized by an increase in the size (hypertrophy) or number (hyperplasia) of adipocytes. The triglyceride (TG) content in adipocytes reflects the balance between lipogenesis and lipolysis, which is largely related to cell volume. When adipocytes reach a critical size threshold, preadipocytes in close proximity to the adipocytes will respond to positive energy balance by proliferating and then differentiating into adipocytes to store the excess energy [1]. Early in life, adipose tissue expansion occurs primarily through hyperplasia. However, humans and rodents have the capacity to form new fat cells from preadipocytes throughout life. Several mechanisms reduce the risk of obesity, including reduced food intake, decreased intestine adsorption, suppressed lipogenesis, enhanced lipolysis and fatty acid oxidation, increased energy expenditure and inhibited preadipocyte proliferation, differentiation, and pharmacological treatment [2,3,4].

*Monascus* species has been used as the traditional food fungus in Eastern Asia for several centuries. *Monascus*-fermented products are gradually developed as the popular and important functional food for the prevention of cardiovascular disease. Red mold dioscorea (RMD) known as the *Monascus purpureus*-fermented *Disocorea batatas* was proven as the strong hypolipidemic functional food in the previous study [5]. However, we found that RMD had only a weak effect on anti-obesity, which limited the development of RMD for the prevention of metabolic syndrome. Monascin and ankaflavin isolated form *Monascus*-fermented product were proven to prevent obesity development via the suppressions of differentiation and lipogenesis in our *in vitro* and *in vivo* studies [5,6]. Therefore, enhancing monascin and ankaflavin levels in RMD may straighten the anti-obesity of RMD.

Deep ocean water (DOW) generally means ocean water from a depth of more than 200 m in depth. The character of DOW includes high purity, cold temperature, abundant nutrients, and minerals [7,8]. Currently, DOW has been applied to food, agriculture, cosmetic and medical field in many countries such as Taiwan, Japan, Korea and America. Due to its high contents of minerals such as magnesium (Mg), calcium (Ca), potassium (K), zinc (Zn), *etc.* [9,10,11,12,13]. A previous study used DOW as the culture water of *Monascus* in order to straighten the hypolipidemic function [14]. DOW-cultured RMD (DOW-RMD) using DOW as the culture water has greater effect on lowering serum total cholesterol (TC), triglyceride (TG), low density lipoprotein cholesterol (LDL-C) levels and raising high density lipoprotein cholesterol (HDL-C) levels than reverse osmosis water-cultured RMD (ROW-RMD). Furthermore, greater anti-atherosclerosis effect, and anti-fatty liver effect are performed by DOW-RMD treatment than ROW-RMD treatment group [14].

According to above-mentioned study, RMD had strong hypolipidemic effect but not anti-obesity effects. RMD cultured in DOW contains substantial amounts of monascin and ankaflavin, and relatively low levels of citrinin. In addition, DOW enhances the production of monascin and ankaflavin. Previous research has shown that monascin and ankaflavin were the effective compounds that perform hypolipidemic and anti-obesity effects. Furthermore, DOW was previously shown to possess anti-obesity capability. Therefore, DOW and the DOW-enhanced functional metabolites in RMD may improve the anti-obesity effect. In this study, animal test was adopted to examine whether DOW-RMD has better anti-obesity effect than ultra-pure water (UPW)-cultured RMD (UPW-RMD) does. Moreover, the effect of ions composition of DOW and DOW-influenced functional metabolites change of RMD on the differentiation and lipogenesis regulation were investigated using 3T3-L1 pre-adipocytes.

## 2. Results

### 2.1. Weight Gain, Food Intake and Feed Efficiency

In the animal test, the rats were randomly assigned to one of the following diets for 8 weeks: standard chow (control group, NOR), high-fat (HF) diet (HF group), HF diet plus 27.81 mg/day 100 g bw UPW-RMD powder (UPW-R-1X group), HF diet plus 27.81 mg/day 100 g bw DOW-RMD powder (DOW-R-1X group), HF diet plus 55.62 mg/day 100 g bw DOW-RMD powder (DOW-R-2X, group), HF diet plus 0.152 mg/day 100 g bw monascin (MS group), HF diet plus 0.162 mg/day 100 g bw ankaflavin (AK group). Following a simultaneous feeding of a HF diet and various test substances, the test animals were sacrificed after 8 weeks and subsequently underwent various analyses. The results are shown in Table 1. The weight gain of the HF group was significantly higher than that of the NOR group (*p* < 0.05), and the weight of the UPW-R1X group did not present significant differences with that of the HF group, indicating that UPW-RMD cannot reduce weight gain. However, the weight gain of the UPW-R1X group was significantly lower than that of the HF group (*p* < 0.05), and the MS group and AK group demonstrated a significant reduction in weight gain (*p* *<* 0.05).

The study results are tabulated in Table 1. Food intake of the HF group was lower than that of the NOR group (*p* < 0.05), because calorie consumption in the HF diet was higher (HF group: 4.17 kcal/g and NOR group: 3.34 kcal/g), and the homeostasis mechanism in the animals caused a reduction in food intake. Food intake of the MS and AK groups were significantly lower than that of the HF group (*p* < 0.05). This result corresponded to that of previous studies [6]. Monascin and ankaflavin may cause the loss of appetite and subsequently reduce food intake. However, further research is required to examine the mechanism.

**Table 1:** Effect of ultra-pure water-cultured-red mold dioscorea (UPW-RMD) and deep ocean water (DOW)-RMD on body weight, food intake and feed efficiency in male Sprague Dawley (SD) rats.

Groups	Initial body weight (g)	Final body weight (g)	Weight gain (g)	Calorie intake (kcal/8 weeks)	Food intake (g)	Feed efficiency (%)
NOR	463.0 ± 33.9 b	579.0 ± 37.6 bc	116.0 ± 20.9 b	5833.3 ± 125.9 d	1746.5 ± 37.7 d	6.8 ± 1.2 a
HF	430.1 ± 37.4 ab	601.4 ± 42.0 c	171.3 ± 18.0 de	5962.7 ± 203.1 bc	1429.9 ± 48.7 bc	12.0 ± 1.4 de
UPW-R-1X	428.5 ± 28.1 ab	605.4 ± 30.2 c	176.9 ± 22.3 e	6106.1 ± 143.9 c	1464.3 ± 34.5 c	12.7 ± 1.5 e
DOW-R-1X	436.9 ± 18.1 ab	591.9 ± 23.3 bc	155.0 ± 12.6 cd	5858.9 ± 175.1 b	1405.0 ± 42.0 b	10.8 ± 0.9 cd
DOW-R-2X	417.5 ± 12.0 ab	565.8 ± 27.4 b	148.3 ± 20.7 c	5956.4 ± 176.4 bc	1428.4 ± 42.3 bc	9.8 ± 1.4 bc
MS	410.3 ± 20.8 a	514.5 ± 23.1 a	104.3 ± 5.2 ab	5262.5 ± 264.0 a	1262.0 ± 63.3 a	7.9 ± 0.6 ab
AK	413.8 ± 17.0 a	512.1 ± 26.9 a	97.4 ± 11.4 a	5447.3 ± 233.1 a	1306.3 ± 55.9 a	6.3 ± 1.2 a

NOR, normal diet (3.34 kcal/g); HF, high-fat diet (4.17 kcal/g); UPW-R-1X, UPW-RMD powder (1×, 27.81 mg/day 100 g bw) and high-fat diet; DOW-R-1X, DOW-RMD powder (1×, 27.81 mg/day 100 g bw) and high-fat diet; DOW-R-2X, DOW-RMD powder (2×, 55.62 mg/day 100 g bw) and high-fat diet; and high-fat diet; MS, monascin powder (2×, 0.152 mg/day 100 g bw) and high-fat diet; AK, ankaflavin powder (2×, 0.162 mg/day 100 g bw) and high-fat diet. Data are presented as means ± SD (*n* = 8). Mean values within each column with different superscripts are significant difference (*p* < 0.05).

Feed efficiency can be regarded as the ability to convert feed mass into increased body mass. According to Table 1, the feed efficiency of the HF group was significantly increased (*p* < 0.05). No significant difference was identified between the feed efficiency of the UPW-R1X group and the HF group (*p* > 0.05). The feed efficiency of the DOW-R1X group substantially decreased because this group was fed with DOW-RMD. Furthermore, the reduction in the feed efficiency of the DOW-R2X group was further enhanced. The feed efficiency of the MS and AK groups also declined significantly (*p* < 0.05). 

In summary, the weight gain and feed efficiency of DOW-RMD declined significantly, possibly because DOW-RMD has higher levels of monascin and ankaflavin than UPW-RMD does. 

### 2.2. Fat Pads Weight

The effects of DOW-RMD on the fat pads weight of rats fed with the HF diet are shown in Table 2. The weights for the total fat pads, perirenal fat pads, and epididymal fat pads of the DOW-R1X group were all lower than those of the UPW-R1X group; however, no significant differences were presented between the two groups (*p* > 0.05). The MS and AK groups demonstrated the ability to significantly reduce the weight of the fat pads, suggesting that DOW can further enhance the effect of reducing total fat pad weight by increasing monascin and ankaflavin levels.

**Table 2:** Effect of UPW-RMD and DOW-RMD on total fat pads, perirenal fat pads and epididymal fat pads weight in male SD rats.

Groups	Total fat pads weight (g)	Perirenal fat pads weight (g)	Epididymal fat pads weight (g)
NOR	16.8 ± 2.6 a	10.4 ± 1.4 ab	8.3 ± 1.5 ab
HF	27.6 ± 3.5 c	17.0 ± 3.9 d	13.0 ± 1.5 d
UPW-R-1X	25.4 ± 7.0 bc	14.9 ± 5.4 cd	10.5 ± 1.9 c
DOW-R-1X	22.8 ± 4.1 b	12.8 ± 2.7 bc	9.8 ± 1.3 bc
DOW-R-2X	18.9 ± 2.2 a	10.7 ± 1.5 ab	8.5 ± 1.2 ab
MS	16.5 ± 2.8 a	9.4 ± 1.6 a	6.9 ± 1.2 a
AK	17.5 ± 2.5 a	9.9 ± 1.4 ab	6.9 ± 1.4 a

NOR, normal diet (3.34 kcal/g); HF, high-fat diet (4.17 kcal/g); UPW-R-1X, UPW-RMD powder (1×, 27.81 mg/day 100 g bw) and high-fat diet; DOW-R-1X, DOW-RMD powder (1×, 27.81 mg/day 100 g bw) and high-fat diet; DOW-R-2X, DOW-RMD powder (2×, 55.62 mg/day 100 g bw) and high-fat diet; and high-fat diet; MS, monascin powder (2×, 0.152 mg/day 100 g bw) and high-fat diet; AK, ankaflavin powder (2×, 0.162 mg/day 100 g bw) and high-fat diet. Data are presented as means ± SD (*n* = 8). Mean values within each column with different superscripts are significant difference (*p* < 0.05).

### 2.3. Cross-Sectional Area and Cell Number of Adipocytes

As shown in Table 3, compared to the UPW-R1X group, the cross-sectional area of the perirenal and epididymal adipocytes of the DOW-R1X group decreased significantly (*p* < 0.05), meaning that DOW-RMD was able to significantly reduce a larger cross-sectional area of adipocytes than the UPW-RMD. Moreover, DOW-RMD significantly reduced a higher number of adipocytes than the UPW-RMD did. In addition, DOW-RMD contains the effective compounds monascin and ankaflavin; therefore, because DOW-RMD comprises higher levels of monascin and ankaflavin, this RMD was able to inhibit the lipogenesis of adipocytes, thereby decreasing the cross-sectional area of adipocytes. 

**Table 3:** Effect of UPW-RMD and DOW-RMD on cell cross-sectional area and cell number of adipocyte in male SD rats.

	Perirenal			Epididymal	
Groups	Cell cross-sectional area (µm^2^)	Cell number (×10^4^)		Cell cross-sectional area (μm^2^)	Cell number (×10^4^)
NOR	16,434 ± 2033 b	7.52 ± 0.88 a		14,555 ± 2766 a	7.40 ± 0.95 a
HF	23,453 ± 3397 c	14.85 ± 2.63 d		22,400 ± 3097 b	13.07 ± 1.33 c
UPW-R-1X	19,283 ± 3455 b	12.87±1.57 cd		19,465 ± 2122 b	10.45± 0.89 b
DOW-R-1X	16,023 ± 2745 a	11.45±0.98 c		15,390 ±1983 a	8.44± 1.45 ab
DOW-R-2X	14,005 ± 2409 a	10.44±1.08 b		14,186 ± 2309 a	7.64± 1.04 a
MS	15,093 ± 3011 a	9.50 ± 1.57 ab		15,123 ± 2793 a	7.28 ± 1.37 a
AK	14,302 ± 2320 a	9.90 ± 1.56 ab		14,907 ± 2123 a	7.44± 1.58 a

NOR, normal diet (3.34 kcal/g); HF, high-fat diet (4.17 kcal/g); UPW-R-1X, UPW-RMD powder (1×, 27.81 mg/day 100 g bw) and high-fat diet; DOW-R-1X, DOW-RMD powder (1×, 27.81 mg/day 100 g bw) and high-fat diet; DOW-R-2X, DOW-RMD powder (2×, 55.62 mg/day 100 g bw) and high-fat diet; and high-fat diet; MS, monascin powder (2×, 0.152 mg/day 100 g bw) and high-fat diet; AK, ankaflavin powder (2×, 0.162 mg/day 100 g bw) and high-fat diet. Data are presented as means ± SD (*n* = 8). Mean values within each column with different superscripts are significant difference (*p* < 0.05).

### 2.4. Lipase Activity and HR-LPL (Heparin-Releasable Lipoprotein Lipase) Activity of Fat Pads

TG hydrolysis of mature adipocytes produces glycerol and free fatty acids, which are then released into extracellular or intracellular spaces to undergo oxidation for energy production or to be employed as raw materials for TG synthesis. Glycerol kinase in adipocytes is present in extremely low quantities; thus, glycerol cannot be reused. Consequently, the glycerol produced from lipolysis is released into extracellular space [5]. The results in Table 4 showed that because the HF group was fed with a relatively high quantity of HF diet, the large amount of adipocytes resulted in the feedback inhibition of adipogenesis and consequently increased lipase activity. The UPW-R1X group has higher lipase activity than HF group, but this activity is higher in the DOW-R1X group. Monascin and ankaflavin are also capable of enhancing lipase activity. Therefore, using DOW as the culture medium increases the of monascin and ankaflavin levels in RMD, thereby improving the ability of RMD to increase lipase activity.

LPL hydrolyzes the TG in the blood lipoprotein, and the products of hydrolysis are then absorbed by surrounding tissues. Therefore, LPL is crucial to adipocytes in that this enzyme facilitates lipid droplet accumulation [5]. According to Table 4, feeding the test animals with DOW-RMD but not UPW-RMD significantly decreased the HR-LPL activity, as compared with HF group. In addition, monascin and ankaflavin are capable of inhibiting HR-LPL activity. Thus, because DOW-RMD may contain comparatively high levels of monascin and ankaflavin, the ability of this RMD to inhibit the HR-LPL activity was significantly enhanced.

**Table 4:** Effect of UPW-RMD and DOW-RMD on lipase activity and Heparin-Releasable Lipoprotein Lipase (HR-LPL) activity of fat pads in male SD rats.

Groups	Lipase activity (U/g fat pad)	HR-LPL activity (U/g fat pad)
NOR	100.0 ± 11.7 a	100.0 ± 25.8 a
HF	140.9 ± 15.1 b	194.6 ± 27.1 c
UPW-R-1X	157.7 ± 13.4 bc	182.8 ± 40.3 bc
DOW-R-1X	165.5 ± 9.4 c	148.0 ± 43.9 ab
DOW-R-2X	168.0 ± 11.2 c	136.7 ± 54.3 ab
MS	169.8 ± 12.1 c	128.1 ± 60.2 ab
AK	165.8 ± 15.0 c	141.2 ± 39.4 ab

NOR, normal diet (3.34 kcal/g); HF, high-fat diet (4.17 kcal/g); UPW-R-1X, UPW-RMD powder (1×, 27.81 mg/day 100 g bw) and high-fat diet; DOW-R-1X, DOW-RMD powder (1×, 27.81 mg/day 100 g bw) and high-fat diet; DOW-R-2X, DOW-RMD powder (2×, 55.62 mg/day 100 g bw) and high-fat diet; and high-fat diet; MS, monascin powder (2×, 0.152 mg/day 100 g bw) and high-fat diet; AK, ankaflavin powder (2×, 0.162 mg/day 100 g bw) and high-fat diet. Data are presented as means ± SD (*n* = 8). Mean values within each column with different superscripts are significant difference (*p* < 0.05).

### 2.5. Blood Lipid

Obesity frequently causes an increase in blood lipid concentration, which increases the risk of cardiovascular disease. Thus, the TC, TG, HDL-C, and LDL-C concentrations are key factors that control metabolic syndrome. The results are presented in Table 5. Because high fat diet does not contain cholesterol, the TC level of the HF group did not increase significantly. Therefore, the TC levels of each group did not present significant differences (*p* > 0.05). The TG level of the HF group increased substantially because this group was provided a HF diet (*p* < 0.05). Previous studies have shown that RMD has the ability to reduce blood lipid, and monascin and ankaflavin were proven as the effective compounds. The results of this study indicated that the TG levels of the DOW-R1X and UPW-R1X groups were significantly lower than that of the HF group (*p* < 0.05). However, the TG level of the DOW-R1X group was significantly lower than that of the UPW-R1X group (*p* < 0.05), and monascin and ankaflavin were able to considerably reduce the TG level (*p* < 0.05). Regarding the HDL-C and LDL-C concentrations, the results indicated that both DOW-RMD and UPW-RMD reduced the LDL-C concentration but not the HDL-C concentration, which consequently achieves the effect of preventing cardiovascular disease. 

### 2.6. Ketone Body and Creatine Kinase Activity

HF diet intake may increase the supply of fatty acids. Free fatty acids are the main ingredient of ketone body (D-3-hydroxybutyrate) synthesis in the liver. Excessive ketone bodies are excreted along with sodium (Na) salt, causing loss of body fluids (*i.e*., dehydration). Subsequently, loss of excessive Na salt results in a pH imbalance and blood acidification, which ultimately leads to complications in the physiological functions of the body. Creatine kinase is an enzyme that catalyzes the conversion of creatine phosphate into creatine to produce energy for muscles. Thus, a significant increase in the level of creatine kinase represents the occurrence of muscle disease [15]. The effects of test substances on the concentration of ketone bodies and creatine kinase activity are shown in Table 6. Each substance was able to substantially reduce the concentration of D-3-hydroxybutyrate. The results of this study indicated that both DOW-RMD and UPW-RMD reduce the D-3-hydroxybutyrate concentration in the blood because these RMD reduce the levels of TG consisting of glycerol and fatty acid. Therefore, the D-3-hydroxybutyrate may be decreased by the decrease of free fatty acid levels. Moreover, monascin and ankaflavin are effective compounds that reduce lipase activity. The creatine kinase activity in the serum was not affected, suggesting that all test substances did not induce muscle damage. 

**Table 5:** Effect of UPW-RMD and DOW-RMD on serum lipidic parameters in male SD rat.

Groups	TC (mg/dL)	TG (mg/dL)	HDL-C (mg/dL)	LDL-C (mg/dL)
NOR	63.1 ± 9.3 a	62.6 ± 10.3 ab	28.3 ± 1.9 bc	24.2 ± 6.2 ab
HF	65.1 ± 8.6 a	94.5 ± 17.5 c	22.6 ± 2.4 a	31.5 ± 7.9 b
UPW-R-1X	63.5 ± 6.9 a	69.8 ± 8.4 b	27.3 ± 3.6 bc	27.2 ± 7.3 ab
DOW-R-1X	64.6 ± 7.9 a	52.8 ± 8.5 a	28.8 ± 1.6 c	25.2 ± 6.6 ab
DOW-R-2X	62.9 ± 2.3 a	60.5 ± 20.1 ab	26.3 ± 2.0 bc	22.0 ± 6.6 a
MS	67.0 ± 16.1 a	67.1 ± 14.5 ab	22.5 ± 3.5 a	24.5 ± 13.2 ab
AK	60.2 ± 6.8 a	60.4 ± 4.6 ab	22.2 ± 1.1 a	18.6 ± 5.4 a

NOR, normal diet (3.34 kcal/g); HF, high-fat diet (4.17 kcal/g); UPW-R-1X, UPW-RMD powder (1×, 27.81 mg/day 100 g bw) and high-fat diet; DOW-R-1X, DOW-RMD powder (1×, 27.81 mg/day 100 g bw) and high-fat diet; DOW-R-2X, DOW-RMD powder (2×, 55.62 mg/day 100 g bw) and high-fat diet; and high-fat diet; MS, monascin powder (2×, 0.152 mg/day 100 g bw) and high-fat diet; AK, ankaflavin powder (2×, 0.162 mg/day 100 g bw) and high-fat diet. Data are presented as means ± SD (*n* = 8). Mean values within each column with different superscripts are significant difference (*p* < 0.05).

**Table 6:** Effect of UPW-RMD and DOW-RMD on serum D-3-hydroxybutyrate, and creatine kinase in male SD rat.

Groups	D-3-hydroxybutyrate (mmole/L)	Creatine kinase (U/L)
NOR	1.86 ± 0.43 b	111.8 ± 55.7 ab
HF	2.06 ± 0.30 b	108.8 ± 67.6 ab
UPW-R-1X	0.46 ± 0.23 a	76.9 ± 19.4 ab
DOW-R-1X	0.31 ± 0.07 a	75.3 ± 27.4 ab
DOW-R-2X	0.57 ± 0.18 a	69.9 ± 16.4 a
MS	0.14 ± 0.04 a	115.7 ± 32.9 b
AK	0.27 ± 0.09 a	107.3 ± 15.8 b

NOR, normal diet (3.34 kcal/g); HF, high-fat diet (4.17 kcal/g); UPW-R-1X, UPW-RMD powder (1×, 27.81 mg/day 100 g bw) and high-fat diet; DOW-R-1X, DOW-RMD powder (1×, 27.81 mg/day 100 g bw) and high-fat diet; DOW-R-2X, DOW-RMD powder (2×, 55.62 mg/day 100 g bw) and high-fat diet; and high-fat diet; MS, monascin powder (2×, 0.152 mg/day 100 g bw) and high-fat diet; AK, ankaflavin powder (2×, 0.162 mg/day 100 g bw) and high-fat diet. Data are presented as means ± SD (*n* = 8). Mean values within each column with different superscripts are significant difference (*p* < 0.05).

### 2.7. Liver and Kidney Function

To determine whether the adoption of test substances to reduce body fat and anti-obesity effect simultaneously causes side effects or complications, we tested the effects of the substances on liver and kidney tissues. The results of relevant index analyses showed that each substance did not significantly influence the liver and kidney (Table 7).

**Table 7:** Effect of UPW-RMD and DOW-RMD on hepatosomatic and renalindex in male SD rat.

Groups	AST (U/L)	ALT (U/L)	Creatinine (mg/dL)	Uric acid (mg/dL)
NOR	113.4 ± 11.5 b	53.8 ± 3.8 ab	0.50 ± 0.00 c	3.1 ± 0.8 a
HF	111.3 ± 14.2 b	52.1 ± 3.9 ab	0.50 ± 0.00 c	4.0 ± 0.8 bc
UPW-R-1X	93.0 ± 10.5 ab	42.8 ± 5.7 b	0.44 ± 0.07 ab	3.6 ± 0.5 ab
DOW-R-1X	85.1 ± 11.0 a	38.6 ± 4.5 a	0.39 ± 0.04 a	3.0 ± 0.5 a
DOW-R-2X	88.8 ± 7.4 a	45.5 ± 2.6 ab	0.43 ± 0.07 ab	3.2 ± 0.3 a
MS	95.9 ± 42.1 ab	63.1 ± 53.1 b	0.44 ± 0.04 ab	4.4 ± 1.0 c
AK	89.3 ± 21.8 ab	51.9 ± 26.0 ab	0.45 ± 0.04 bc	4.5 ± 0.4 c

NOR, normal diet (3.34 kcal/g); HF, high-fat diet (4.17 kcal/g); UPW-R-1X, UPW-RMD powder (1×, 27.81 mg/day 100 g bw) and high-fat diet; DOW-R-1X, DOW-RMD powder (1×, 27.81 mg/day 100 g bw) and high-fat diet; DOW-R-2X, DOW-RMD powder (2×, 55.62 mg/day 100 g bw) and high-fat diet; and high-fat diet; MS, monascin powder (2×, 0.152 mg/day 100 g bw) and high-fat diet; AK, ankaflavin powder (2×, 0.162 mg/day 100 g bw) and high-fat diet. Data are presented as means ± SD (*n* = 8). Mean values within each column with different superscripts are significant difference (*p* < 0.05).

### 2.8. Electrolyte Balance

The composition and concentration of electrolytes in the body fluids must be maintained at a certain level to stabilize body pH, osmotic pressure, and water balance, as well as to sustain normal cell growth. Acting as an indicator for electrolyte balance analysis, Na^+^ and K^+^ are cations that are found in the highest quantity inside and outside of cells, respectively. As shown in Table 8, each test substance does not affect the electrolyte balance.

**Table 8:** Effect of UPW-RMD and DOW-RMD on electrolyte balance in male SD rat.

Groups	Na (mEq/L)	K (mEq/L)
NOR	146.8 ± 1.5 a	6.7 ± 0.5 bc
HF	149.4 ± 0.4 b	6.7 ± 0.6 bc
UPW-R-1X	148.8 ± 1.3 b	7.0 ± 0.7 bc
DOW-R-1X	149.5 ± 1.3 b	6.5 ± 0.3 ab
DOW-R-2X	149.7 ± 0.8 b	6.4 ± 0.6 ab
MS	148.9 ± 1.1 b	7.2 ± 0.6 c
AK	149.0 ± 1.1 b	7.2 ± 0.3 c

NOR, normal diet (3.34 kcal/g); HF, high-fat diet (4.17 kcal/g); UPW-R-1X, UPW-RMD powder (1×, 27.81 mg/day 100 g bw) and high-fat diet; DOW-R-1X, DOW-RMD powder (1×, 27.81 mg/day 100 g bw) and high-fat diet; DOW-R-2X, DOW-RMD powder (2×, 55.62 mg/day 100 g bw) and high-fat diet; and high-fat diet; MS, monascin powder (2×, 0.152 mg/day 100 g bw) and high-fat diet; AK, ankaflavin powder (2×, 0.162 mg/day 100 g bw) and high-fat diet. Data are presented as means ± SD (*n* = 8). Mean values within each column with different superscripts are significant difference (*p* < 0.05).

### 2.9. Monascin and Ankaflavin Production

To determine whether the main ions in the DOW enhance monascin and ankaflavin production and inhibit citrinin formation, this study prepared the synthetic water (SW) according to the composition and concentration of main ions in DOW, which was then used to culture *Dioscorea batatas* to produce the SW-cultured RMD (SW-RMD). The analytical results for the monascin, ankaflavin, and citrinin levels showed that DOW-RMD and SW-RMD have significantly higher levels of monascin than UPW-RMD, and SW-RMD has a considerably lower level of ankaflavin than DOW-RMD (Table 9). Furthermore, SW significantly reduced the citrinin level in RMD. Based on the above findings, additional ions that possess the ability to boost monascin and ankaflavin productions may be present in DOW. Moreover, other ions are possibly present to antagonize the reduction of citrinin formation, consequently allowing DOW to reduce the level of citrinin (*i.e.*, compared to SW). 

**Table 9:** Effect of UPW, DOW, and synthetic water (SW) on the production of monascin, ankaflavin, citrinin in RMD.

Groups	Monascin concentration (mg/kg)	Ankaflavin concentration (mg/kg)	Citrinin concentration (mg/kg)
UPW-RMD	2146 ± 35 a	2221 ± 4 a	2.864 ± 0.085 b
DOW-RMD	2727 ± 219 b	2912 ± 263 b	2.725 ± 0.090 b
SW-RMD	2614 ± 175 b	2414 ± 176 c	2.312 ± 0.092 a

UPW-RMD, the red mold dioscorea cultured by ultra-pure water; DOW-RMD, the red mold dioscorea cultured by deep ocean water; SW-RMD, the red mold dioscorea cultured by synthetic water; Data are presented as means ± SD (*n* = 3). Mean values within each column with different superscripts are significant difference (*p* < 0.05).

### 2.10. Differentiation of Pre-Adipocytes

Based on the above-mentioned results, DOW-RMD possessed a superior ability of reducing weight and body fat. In this study, to understand how DOW-RMD regulates body fats metabolism, the effects of DOW-RE on adipocyte differentiation and lipogenesis were investigated using *in vitro* cell tests. In addition, ions in DOW may possibly influence adipocyte regulation; thus, in a cell model, we simulated an SW containing the ions in DOW. The SW contained Mg^2+^, Ca^2+^, Na^+^, K^+^, Fe^2+^, and Zn^2+^, which are the main ions of DOW, and the concentrations of these ions was identical to those in DOW. The objective was to investigate how the main ions of DOW alter the ability of RMD for adipogenesis regulation, and to elucidate whether these ions are crucial in DOW.

**Figure 1:**
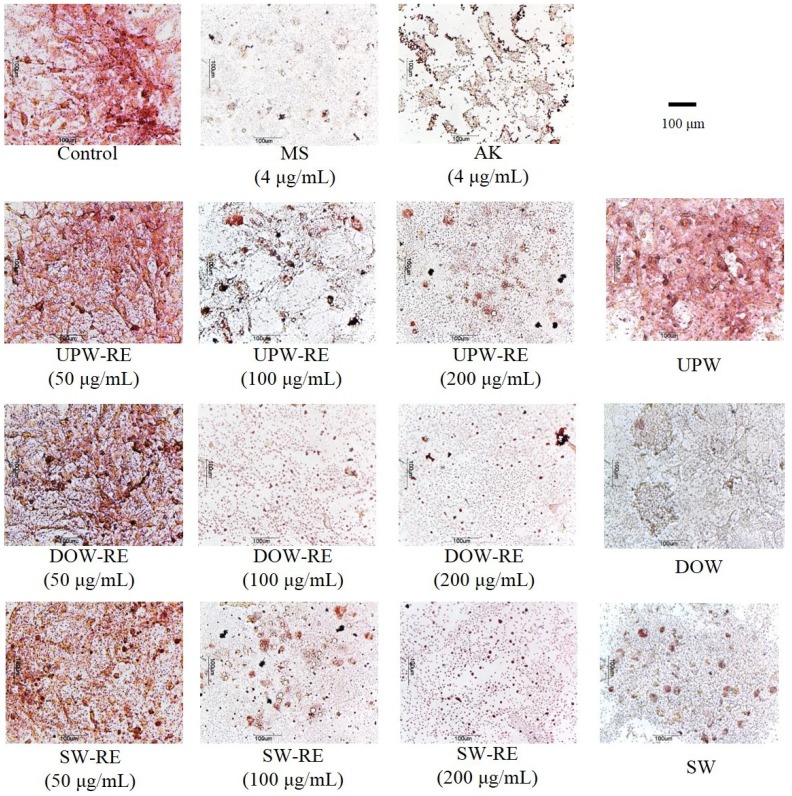
Effects of DOW-RMD ethanol extracts (DOW-RE), UPW-RMD ethanol extracts (UPW-RE), SW-RMD ethanol extracts (SW-RE), monascin (MS), ankaflavin (AK) on 3T3-L1 preadipocyte differentiation. Preadipocytes were differentiated according to the method described in ‘Materials and methods.’ During differentiation the cells were treated with various samples. On day 8, the cells were fixed and stained with oil-red O.

Following the ninth day of induced differentiation, 3T3-L1 pre-adipocyte underwent oil-red O staining, in which the dye combined with neutral oil to form red oil droplets that facilitated the observation of cell differentiation. According to the experimental results (Figure 1), monascin and ankaflavin effectively inhibited the accumulation of lipid droplets in cells. Moreover, 100 μg/mL and 200 µg/mL of UPW-RE, DOW-RE, and SW-RE extracts effectively and significantly inhibited the accumulation of lipid droplets (*p* < 0.05). Particularly, at 100 μg/mL of DOW-RE, the amount of lipid droplets accumulated was significantly lower than that at 100 μg/mL of UPW-RE and 100 μg/mL of SW-RE, respectively. The results suggested that the use of UPW, DOW, and SW to culture RMD effectively inhibited differentiation of pre-adipocytes, and DOW-RE produced better results possibly because DOW has higher levels of monascin and ankaflavin.

Subsequently, we examined whether DOW possesses inhibitory effects (Figure 1). Adding DOW and SW effectively inhibited the accumulation of adipocyte lipid droplets. In addition, DOW demonstrated better inhibitory effect than SW, indicating that DOW may be the primary cause of enabling DOW-RE to inhibit lipid droplet accumulation. Furthermore, the main ions selected from SW may be extremely crucial in facilitating the reduction of lipid droplet formation. 

### 2.11. Transcription Factor Expression during Differentiation

Differentiation agents were added to pre-adipocytes to induce differentiation. Subsequently, key transcription factors were activated to enhance adipocyte maturation, which increased protein expression and consequently stimulates lipid droplet accumulation. On the second day of differentiation, we examined whether the test substances were able to effectively inhibit the protein expression of transcription factor C/EBPβ. Furthermore, on the sixth day of differentiation, changes in the protein expression of transcription factors PPARγ and C/EBPα were investigated. Because C/EBPβ is activated at the initial stage of differentiation, it is expressed briefly, thereby activating the expression of PPARγ and C/EBPα. Thus, protein expressions of the three key transcription factors were analyzed to explore the effects of DOW and the products of RMD on differentiation in this study.

**Figure 2:**
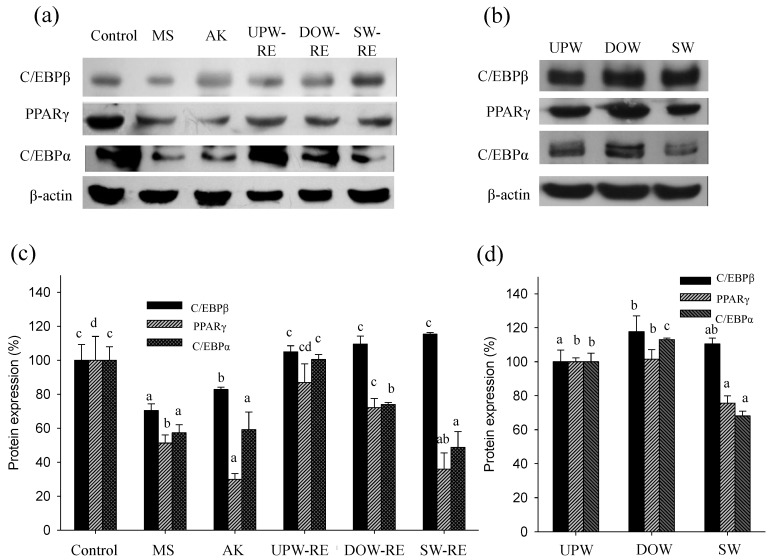
Effect of various substances on C/EBPβ, PPARγ, and C/EBPα protein expressions in 3T3-L1 preadipocyte differentiation. (**a**) Target protein expressions were visualized using immunoblotting in the treatment of DOW-RMD ethanol extracts (DOW-RE), UPW-RMD ethanol extracts (UPW-RE), SW-RMD ethanol extracts (SW-RE), monascin (MS), and ankaflavin (AK); (**b**) Target protein expressions were visualized using immunoblotting in the treatment of UPW, DOW, and SW. (**c**) Quantification of protein expressions in the treatment of DOW-RE, UPW-RE, SW-RE, MS, and AK. (**d**) Quantification of protein expressions in the treatment of UPW, DOW, and SW. Mean values with different superscripts are significant difference (*p* < 0.05).

As shown in Figure 2a,c, using monascin and ankaflavin treatment, the C/EBPβ protein expression for the MS and AK groups was significantly reduced (*p* < 0.05). However, significant lowering effects in the UPW-RE, DOW-RE, and SW-RE treatments were not observed. The experimental results showed that each extract solution was unable to significantly reduce C/EBPβ expression, possibly because the monascin level was insufficient to trigger inhibition. Subsequently, we determined whether DOW can effectively enhance effect of RMD on inhibiting PPARγ expression. The results for the PPARγ protein expression on the sixth day of differentiation are shown in Figure 2a,c. Monascin and ankaflavin treatments significantly lowered the PPARγ protein expression (*p* < 0.05). PPARγ protein expression were also reduced to 86.95%, 72.14%, and 35.99% by the treatments of UPW-RE, DOW-RE, and SW-RE, respectively (*p* < 0.05). Furthermore, the presence of monascin and ankaflavin significantly reduced the expression of C/EBPα protein to 57.34% and 59.22%, respectively (*p* < 0.05). Although the UPW-RE extract did not influence C/EBPα expression, the DOW-RE and SW-RE treatments decreased the expression to 74.01% and 48.80%, respectively (*p* < 0.05).

Overall, DOW-RE possessed better ability than UPW-RE to reduce the expression of the transcription factors. Therefore, this study next determined whether DOW and SW affected expression of the transcription factors. The results showed that DOW treatment did not significantly influence the expression of C/EBPβ, PPARγ, and C/EBPα; however, SW treatment was able to inhibit PPARγ, and C/EBPα expressions (Figure 2b,d). Summarizing the above results showed that DOW-RE effectively inhibited the expressions of the transcription factors compared to UPW-RE. Because SW-RE (in contrast to DOW-RE and UPW-RE) was able to substantially inhibit the expressions of the transcription factors, we speculated that besides monascin and ankaflavin, the main ions in DOW enabled DOW-RMD to possess better ability of inhibiting differentiation. 

### 2.12. Lipolysis Effect and HR-LPL Activity in Lipogenesis

**Figure 3:**
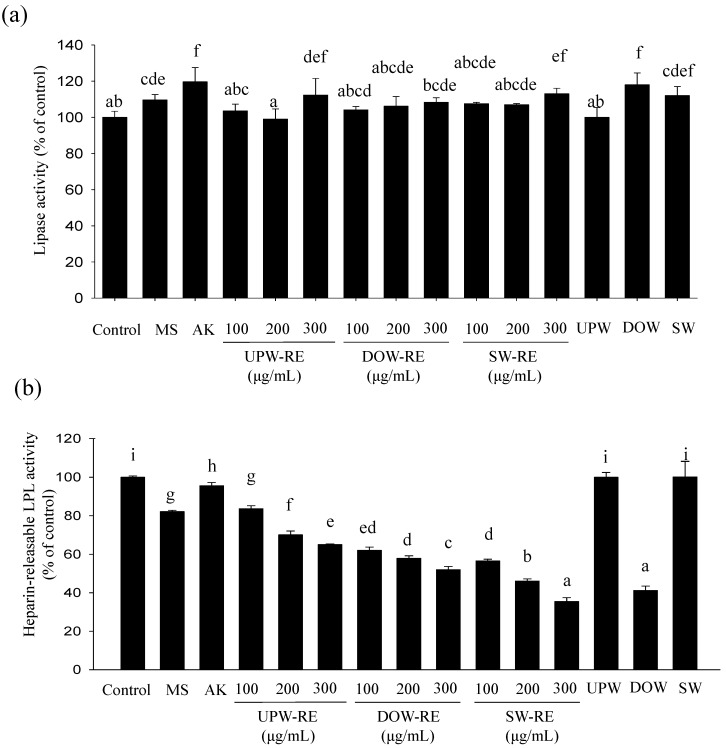
Effect of DOW-RMD ethanol extracts (DOW-RE), UPW-RMD ethanol extracts (UPW-RE), SW-RMD ethanol extracts (SW-RE), monascin (MS), ankaflavin (AK), UPW, DOW, SW on lipogenesis in mature 3T3-L1 adipocyte. (**a**) lipase activity (**b**) HR-LPL activity. Mean values with different superscripts are significant difference (*p* < 0.05).

After 12 days of differentiation, the majority of 3T3-L1 pre-adipocytes have transformed to mature adipocytes. The lipolysis effect was investigated. As shown in Figure 3a, after monascin and ankaflavin treatments, the lipolysis effect were increased to 110% and 120%, respectively, showing a significant difference (*p* < 0.05); however, the extent of the increase was unsubstantial. The RMDs cultured with three types of water presented a weak lipolysis effect. Furthermore, the lipolysis effect in DOW-RE and SW-RE treatments were increased to 118% and 112%, respectively, but the extent of the increase was not significant (*p* > 0.05). Therefore, both DOW-RE and SW-RE demonstrated a weak lipolysis effect.

HR-LPL is a crucial enzyme that influences the lipogenesis of mature adipocytes and stimulates the accumulation of fatty acid to form lipid droplets. The effects of the test substances on the activity of HR-LPL are presented in Figure 3b. Monascin treatment was shown to significantly reduce the LPL activity to 82% (*p* < 0.05). Moreover, treatments with 100 μg/mL of UPW-RE, DOW-RE, and SW-RE reduced the HR-LPL activity to 84%, 62%, and 57%, respectively (*p* < 0.05), and significant differences among the three REs were observed (*p* < 0.05). Therefore, DOW-RE and SW-RE all presented better ability in inhibiting HR-LPL activity; particularly, SW-RE has better inhibition ability than DOW-RE. Furthermore, DOW significantly reduced HR-LPL activity to 41%, whereas SW has no effect on the activity. Based on the results, DOW possessed an inhibitory effect, and DOW-RE demonstrated a better inhibitory effect on the LPL activity. These results indicated that DOW may inhibit lipid droplet formation by employing its functional effects and enhancing monascin production in RMD. However, the main ions in SW do not possess the ability to inhibit LPL activity. 

## 3. Discussion

DOW, enriched with minerals and trace elements, has been applied in the manufacturing of various fermented food products, such as sake, soya sauce, and miso. Moreover, DOW is composed many major elements, Mg^2+^, Ca^2+^, K^+^, Zn^2+^, Fe^2+^, *etc*., that enhances the secondary metabolite production of *Monascus purpureus* [14]. In this study, DOW and UPW were used to culture RMD. The analytical results indicated that DOW-cultured RMD contains a relatively high level of monascin and comparatively low amount of citrinin. Monascin yield increased by 22%, and citrinin yield was reduced by 33%, suggesting that certain minerals or trace elements in DOW may enhance *Monascus* to produce higher amounts of monascin and inhibit the production of citrinin [14]. However, these salts such as NaCl, MgCl_2_, KCl, and *etc.* in DOW may also induce stress due to decreased water availability (water activity). The previous studies indicated that the growth and secondary metabolites formation of fungus were stimulated by the stress [16,17]. Furthermore, the previous studies indicated that salts have a major impact on the nature and extent of the biosphere, because solutes radically influence water activity and exert other activities that also affect biological systems (e.g., ionic, kosmotropic, chaotropic and those that affect cell turgor), and as a consequence can be major stressors of cellular systems. MgCl_2_ known as a chaotropic agent disrupts the structure of macromolecules such as proteins and nucleic acids. The extreme chaotropicity of MgCl_2_ at high concentrations not only denatures macromolecules, but also preserves the more stable ones: such indicator molecules, hitherto regarded as evidence of life, may thus be misleading signatures in chaotropic environments [18,19,20]. Compared to DOW, UPW has a high water activity that is supra-optimal and stressful for growth and metabolism of fungus [16,21]. Therefore, DOW including salts may act a stressor with lower water activity that may cause up-regulation of growth, secondary metabolites production, and pharmaceutical activities [21,22,23].

Regarding the reduction of body-fats, compared to UPW-RMD, DOW-RMD possessed better ability of lowering weight gain, food intake, feed efficiency, body-fat pad, cross-sectional area of adipocytes, serum TG level, and liver TC level. Previous research has confirmed that *Monascus* was able to reduce body-fats; however, the ability of RMD to reduce body-fat was not significant [24]. In this study, DOW-RMD, but not UPW-RMD, demonstrated the ability to significantly reduce weight gain, body-fat pad, and the cross-sectional area of adipocytes. This result suggested that DOW use in RMD fermentation can enhance the anti-obesity effect of RMD. 

Numerous factors explain the reason that DOW contributed higher anti-obesity to DOW-RMD, one of which is that the water absorbed during the fermentation is retained in RMD, which therefore improved the anti-obesity function in DOW-RMD. According to a previous study, drinking DOW reduces the PPAR-γ expression in adipocytes [12]. Furthermore, in an *in vitro* cell test, Hwang *et al.* (2009) [25] investigated the mechanism of how DOW influences 3T3-L1 pre-adipocytes. The results verified that DOW effectively inhibited the proliferation of pre-adipocytes and significantly reduced the accumulation of lipid droplets. Moreover, DOW was able to reduce the regulation of adipocyte transcription factors; consequently, differentiation of pre-adipocytes was inhibited.

The other factor may be attributed to the DOW-stimulated functional metabolites production in the RMD. In the previous study, monascin and ankaflavin were observed to inhibit differentiation of 3T3-L1 pre-adipocytes. Specifically, the expressions of C/EBPα, C/EBPβ, C/EBPδ, and PPARγ proteins were inhibited, which also reduced the accumulation of intracellular TG. Moreover, monascin and ankaflavin enhanced the decomposition of intracellular lipid droplets in mature adipocytes lipogenesis to release glycerol and reduce extracellular LPL activity, which subsequently reduces intracellular TG synthesis [5]. In addition to inhibiting mature adipocyte lipogenesis by lowering HR-LPL activity and increasing the lipolysis effect, monascin and ankaflavin were also observed to inhibit pre-adipocyte differentiation in animals by inhibiting the expression of C/EBPα, C/EBPβ, and PPARγ proteins [5].

In the *in vivo* animal test, the results showed that DOW-RMD demonstrated better anti-obesity ability. The ions in DOW may influence the regulation of adipocytes. Therefore, we further simulated SW that contains the major ions in a DOW. In metabolite production, DOW-RMD and SW-RMD are RMDs cultured in DOW and SW. The two products contained similar amount of monascin, but the ankaflavin level in SW-RMD was less than that in DOW-RMD. Thus, the ions in SW are crucial to enhancing monascin production; however, other effective ions in DOW may be present to facilitate an increase in ankaflavin. The levels of monascin and ankaflavin may influence the anti-obesity ability such as differentiation and lipogenesis. The *in vitro* results showed that the inhibitory effect of DOW-RE and SW-RE on PPARγ and C/EBPα protein expressions was substantially better than that of UPW-RE. The inhibitory effect of monascin and ankaflavin on PPARγ and C/EBPα expressions was also evident. Therefore, DOW-RE and SW-RE, compared to UPW-RE, were able to significantly inhibit differentiation possibly because of the relatively high levels of monascin and ankaflavin. Although previous studies have shown that DOW inhibited the expression of C/EBPβ, PPARγ, and C/EBPα [25], the inhibitory effect of DOW on transcription factor expressions was not identified in this study. However, the six ions in SW possessed the ability to inhibit PPARγ and C/EBPα expressions, indicating that these ions are effective in inhibiting differentiation. Besides the six ions contained in DOW, additional ions may exist in small quantities. These additional ions may stimulate PPARγ and C/EBPα expressions. Consequently, although SW-RE contains fewer amounts of monascin and ankaflavin than DOW-RE, its ability to inhibit PPARγ and C/EBPα expressions is superior to DOW-RE. This phenomenon suggests that SW-RMD may have absorbed the ions in SW, which subsequently increased the inhibitory effect on PPARγ and C/EBPα expressions.

In the process of forming lipid droplets in mature adipocytes, the results showed that the lipolysis effect of RMD extract improves with increasing concentration to inhibit lipogenesis. The lipolysis effect of UPW-RE, DOW-RE, and SW-RE demonstrated no significant differences. This result was similar to that obtained in animal testing, where no significant differences were observed between the lipolysis effect of DOW-RMD and UPW-RMD. This observation showed that DOW cannot significantly enhance the lipolysis effect in RMD. This research and previous studies have verified that the lipolysis ability of monascin and ankaflavin [6]. However, increases in monascin and ankaflavin cannot reflect the enhancement of the lipolysis effect in DOW-RMD in this study, possibly because insufficient amounts of monascin and ankaflavin were available to perform lipolysis effect or because monascin and ankaflavin have reached maximum utility that increasing the level does not significantly influence the lipolysis effect. In this study, we identified that significant lipolysis effect was performed by DOW treatment but not DOW-RMD because the amount of DOW absorbed in DOW-RMD may be not more enough to achieve the lipolysis effect.

In this study, as the concentration increased, the RMD extract effectively inhibited LPL activity, thereby inhibiting lipid droplet formation. Compared to the UPW-RE, the DOW-RE was shown to perform a better inhibitory effect on LPL activity. This result corresponded to that of the animal testing, in which DOW-RMD presented a better inhibitory effect on LPL activity. LPL expression is mediated by the activation of PPARγ by cognate ligands, as LPL is a downstream gene of PPARγ. The PPARγ/RXR complex binds to the PPRE present in the promoter region of the LPL gene and increases LPL gene expression [26]. The induction of lipoprotein lipase synthesis by PPARγ is mainly in the mature adipocytes in order to increase local generation of free fatty acids [27,28]. However, monascin is proven to inhibit PPARγ expression and HR-LPL activity in this study and our previous study [6]. Therefore, this could be one of the primary factors of monascin- and ankaflavin-mediated inhibition of lipogenesis. According to the *in vivo* and *in vitro* tests results, more potent LPL activity may be contributed from the higher monascin levels, as well as the absorption of DOW in DOW-RMD. Furthermore, SW-RE but not SW demonstrated an inhibitory effect on LPL activity, suggesting that the LPL activity-lowering effect of SW-RE should be contributed form the increased monascin levels or other compound but not the ions of SW. However, besides the six ions in SW, additional ions in DOW may be the functional ions for the LPL activity-lowering effect. 

SW contains Mg^2+^, Ca^2+^, Na^+^, K^+^, Fe^2+^, and Zn^2+^, all of which are the main ions in DOW. In addition, the concentrations of these ions in SW were adjusted to that in DOW. Although SW contains fewer types of ions than DOW (which comprises more than ten types of ions), it still enhanced the rate of monascin and ankaflavin production in RMD. However, the ability of SW to increase the ankaflavin level is less effective than that of DOW. Despite having several similarities with DOW in monascin production, the ability of DOW and SW in the regulation of adipogenesis exhibits numerous differences. SW is able to inhibit PPARγ and C/EBPα expressions and is able to slightly increase lipolysis effect but not inhibit the HR-LPL activity. Conversely, DOW cannot inhibit PPARγ and C/EBPα expressions but is able to enhance the lipolysis effect and inhibit HR-LPL activity. However, both DOW-RMD and SW-RMD are able to inhibit PPARγ and C/EBPα expressions and improve HR-LPL activity because of the different reason as follow: DOW-RMD contains higher monascin and ankaflavin levels as well as DOW accumulation; and SW-RMD contains higher level of monascin and accumulates six types of effective ions.

Regarding the reduction of blood lipids, in comparison to UPW-RMD, DOW-RMD was able to reduce the level of TC and LDL-C in the serum. This result corresponded to that obtained in our previous results [14]. DOW-RMD performed better blood lipid-reduction effect because it contained higher levels of blood lipid-reducing substances monascin and ankaflavin. Furthermore, DOW comprised minerals and trace elements such as Mg^2+^, Ca^2+^, and K^+^. Numerous studies have indicated that a higher Mg/Ca ratio facilitates the prevention of cardiovascular disease [29]. Cohen *et al.* (2002) showed that an intake of 8.3 g Mg salt per day significantly reduced TC and TG levels in the blood [29]. Shahkhalili *et al.* (2001) [30] identified that supplementing participants with 0.9 mg/day Ca reduced the level of LDL-C in the blood. DOW-RMD was used as the test substance in this study. DOW including Mg^2+^, Ca^2+^, Zn^2+^, Fe^2+^, K^+^, and *etc.* was supplemented daily to culture RMD, accumulated gradually in DOW-RMD; thus, the hypolipidemic effect of was enhanced.

## 4. Experimental Section

### 4.1. Chemicals

LC grade acetonitrile, chloroform, methanol, and dimethyl sulfoxide (DMSO) were purchased from Merck Co. (Darmstadat, Germany). Tryptone, yeast extract, peptone, malt extract, potato dextrose agar (PDA), and Bacto-agar were purchased from Difco Co. (Detroit, MI, USA). Monoclonal C/EBPα antibody was purchased from GeneTex Co (Irvine, CA, USA). Monoclonal C/EBPβ antibody and polyclonal PPARγ antibody were purchased from Novus Biological (Littleton, CO, USA). Dulbecco’s modified Eagle’s medium and fetal bovine serum were purchased from Invitrogen Life Technologies (Carlsbad, CA, USA). Dexamethasone, isobutylmethylxanthine, insulin, oil-red O, heparin, *p*-nitrophenyl butyrate were purchased from Sigma Chemical Co. (St Louis, MO, USA). Trypan blue stain was purchased from Gibco BRL Life Technologies Inc. (Gaithersburg, MD, USA).

### 4.2. The Source of DOW and the Preparation of SW

The DOW purchased from the Taiwan Yes Deep Ocean Water Co. (Hualien, Taiwan) was pumped from a depth of 670 m in the Pacific Ocean near the Eastern Taiwan and processed though the electrodeionization. The concentrations of the trace elements and minerals in DOW including Al, Cu, Zn, As, Ba, Cd, Cr, Pb, Hg, Se, Ag, Ca, Mg, K, Na, Sb, Tl, Be, Fluoride, Nitrate, Sulfate, Chloramines, and Chlorine have been measured and published in our previous study [14]. SW was prepared by mixing the main ions of DOW including 20.65 mg/L Mg, 5.02 mg/L Ca, 7.71 mg/L Na, 0.22 mg/L K, 0.0062 mg/L Fe, and 0.019 mg/L Zn ion with equal concentrations to that in DOW. 

### 4.3. Preparation of UPW-RMD, DOW-RMD, and SW-RMD

*Monascus purpureus* NTU 568 fermented product has been proven to perform a potent hypolipidemic effect in our previous study [31,32]. The culture strain was maintained on PDA slant at 10 °C and transferred monthly. The *Dioscorea* root (*Dioscorea batatas* Dence) purchased from a local supermarket in Taiwan was used to produce RMD using the method of solid-state culture. UPW, DOW, and SW were used as all of the water used in the production of UPW-RMD, DOW-RMD, and SW-RMD, respectively. Five hundred grams *Dioscorea* substrates were, respectively, soaked in distilled water for 8 and 1 h. After that, excess water was removed with a sieve. The substrate was autoclaved for 20 min at 121 °C in a “koji-dish” (the koji-dish is made of wood with the dimension of 30 × 20 × 5 cm). After being cooled, the substrate was inoculated with a 5% (v/w) spore suspension (10^7^ spores/mL). The inoculated substrate was cultured at 28 °C for 10 days. In addition, during the culturing stage, 100 mL of water is added once every 12 h at a total of three times and the addition of water starts on the fifth day of culture. After fermentation, the crushed and dried product with the mold was used for the experiments [14,33]. 

### 4.4. Animal Experiments

Animal experiments protocol is refer to our previous study involved the anti-obesity evaluation of RMR [6]. Male Sprague Dawley (SD) rats at 6–8 weeks of age were purchased from the BioLasco Co. (Taipei, Taiwan). The animals were housed individually and allowed free access to a standard laboratory chow (Ralston Purina, St Louis, MO, USA) and water. Three weeks later, the rats were randomly assigned to one of the following diets for 8 weeks: standard chow (control group, NOR; 4.5% fat, 3.34 kcal/g), high-fat (HF) diet consisting of 26.7% butter powder (Gene Asia Biotech Co., Ltd., Nang-Tou, Taiwan) in standard chow (HF group; 30% fat, 4.17 kcal/g), HF diet plus 27.81 mg/day 100 g bw UPW-RMD powder (UPW-R-1X group), HF diet plus 27.81 mg/day 100 g bw DOW-RMD powder (DOW-R-1X group), HF diet plus 55.62 mg/day 100 g bw DOW-RMD powder (DOW-R-2X, group), HF diet plus 0.152 mg/day mg/day 100 g bw monascin (MS group), HF diet plus 0.162 mg/day 100 g bw ankaflavin (AK group), The recommendation dosage of DOW-RMD or UPW-RMD for anti-obesity effect is suggested as 2 g/day for human in our previous study [24]. The dosage of monascin and ankaflavin in the MS and AK groups were equal to that in the DOW-R-1X group. The doses of the test substances used in this study were calculated according to Boyd’s formula for body surface area for adult humans (weight: 65 kg; height: 170 cm). Each sample was orally administrated to the rats by stomach tube in each group [24]. 

Food consumption and body weight were recorded weekly. At the end of the study, the rats were deprived of food for 16 h before being scarified by CO_2_ asphyxiation. Blood samples were collected from the posterior vena cava and centrifuged at 700× *g* for 10 min; the serum was stored at −20 °C until analyzed. Perirenal and epididymal fat pads were removed and weighed. Portions of the adipose tissue were immersed in 10% formaldehyde for histological inspection; other portions were frozen immediately in liquid nitrogen and stored at −80 °C for analysis of lipolysis and HR-LPL activity. Liver was excised and stored at −20 °C for the measurement of lipids. The experiment was reviewed and approved by the Animal Care and Research Ethics Committee of the National Taitung University.

### 4.5. Biochemical Analyse

The serum total cholesterol (TC), triglyceride (TG), low density lipoprotein cholesterol (LDL-C), high density lipoprotein cholesterol (HDL-C), creatinine, uric acid, Na, K, ketone body (hydroxybutyrate) concentrations, and aspartate aminotransferase (AST), alanine aminotransferase (ALT), and creatine kinase (CK) activities were measured using the commercial kits (Randox Laboratories Ltd., Antrim, UK). Lipolysis effect and HR-LPL activity assay followed the method of our previous studies [6]. 

### 4.6. Adipose Tissue Histology

The adipose tissue samples were fixed in formaldehyde, embedded in paraffin, cut into 5-mm sections and stained with hematoxylin and eosin. Cross-sectional areas of the adipocytes were calculated from the histogram according to Chen and Farese [34]. For the estimation of fat pads cell number, the lipid content of 0.3 g of fat tissue was extracted by using the method of Folch *et al.* [35]. The total cell number in the fat pads was calculated by dividing the lipid content of the fat pad by the mean weight of cell lipids. The lipid weight of the average fat cell was calculated from the mean cell volume, assuming a lipid density of 0.915 (density of triolein).

### 4.7. Cell Culture

3T3-L1 preadipocytes purchased from the Bioresource Collection and Research Center (Hsinchu, Taiwan) were cultured in Dulbecco’s modified Eagle’s medium (DMEM) containing 10% fetal bovine serum) at 37 °C in 5% CO_2_. To induce differentiation, 2-day postconfluent 3T3-L1 preadipocytes (day 0) were stimulated for 48 h with 0.5 mM isobutylmethylxanthine, 1 mM dexamethasone and 10 mg/mL insulin (MDI) added to basal medium. On day 2, the MDI medium was replaced with basal medium containing insulin only. On day 4 and thereafter, the cells were cultured in basal medium, which was freshly changed every 2 days until the cells were analyzed. 

DOW-RE, SW-RE, and UPW-RE were prepared using the extraction of DOW-RMD, SW-RMD, and UPW-RMD with 10-fold volume of 95% ethanol at 37 °C for 24 h, respectively. DOW-RE, SW-RE, and UPW-RE were diluted to various concentrations with DMEM medium, and further used as the treatment medium in the cell experiments. The vehicle control was 0.3% ethanol in culture medium, which was equal to the ethanol concentration in all RMD extract treatments.

### 4.8. Oil-Red O Staining

Differentiated 3T3-L1 cells on day 8 were fixed with 10% formaldehyde and then stained with oil-red O. Pictures were taken using a microscope (ECLIPSE TS100; Nikon Co., Tokyo, Japan) [36].

### 4.9. Lipolysis Assay

The fully differentiated 3T3-L1 adipocytes (days 8–12 after differentiation induction) were treated with test substances in Krebs Ringer bicarbonate (KRB) buffer (20 mM NaCl, 4.7 mM KCl, 2.2 mM CaCl_2_, 1.2 mM MgSO_4_ 7H_2_O, 1.2 mM KH_2_PO_4_, 25 mM NaHCO_3_ and 2% BSA; pH 7.4) for 24 h. Adipose explants (0.1 g) of perirenal and epididymal fat pads from experimental rats were incubated in 1 mL of KRB buffer at 37 °C for 1 h [37]. Glycerol was determined enzymatically from the supernatant by using a Randox kit.

### 4.10. Heparin-Releasable Lipoprotein Lipase (HR-LPL) Activity Assay

After incubation of the 3T3-L1 mature adipocytes with the experimental medium for 24 h, the medium was discarded. The cells were rinsed with KRB buffer and then cultured in heparin-KRB (10 U/mL heparin) at 37 °C for 1 h. The conditioned heparin-KRB was collected from each well for the assay of HR-LPL activity. In the animal study, a sample of perirenal and epididymal adipose tissue weighing 0.1 g was placed in 1 ml of KRB buffer supplemented with 10 U/mL heparin at 37 °C for 1 h. LPL activity was measured according to the previous study on the basis of its esterase property using *p*-nitrophenyl butyrate as a substrate [36].

The TG hydrolase activity of LPL with synthetic TG substrates is inhibited by molar sodium chloride, and this property has been used to distinguish LPL activity from the activities of other lipases in plasma. Thus, HR-LPL activity was calculated from the productivity of *p*-nitrophenol using the following equation [36].

*C* (µM) = [*A*_400_ (0.15 M NaCl) − *A*_400_ (1 M NaCl)]/0.012

where *A*_400_ (0.15 M NaCl) and *A*_400_ (1 M NaCl) were the absorbances of released *p*-nitrophenol at 400 nm in 0.15 M and in 1 M NaCl assay buffer, respectively, and 0.012 is the micromolar extinction coefficient of *p*-nitrophenol.

### 4.11. Immunoblotting

Protein concentration was determined by bicinchoninic acid (BCA) method. A total of 40 μg of total protein from each sample was applied for Western blot representative of three independent experiments according to the previous studies [38,39]. The samples were separated on 10% SDS-PAGE gels and transferred to polyvinylidene fluoride membranes. After blocking in a gelatin-NET solution, blots were incubated with monoclonal C/EBPα antibody (1:5000), monoclonal C/EBPβ antibody (1:2000), polyclonal PPARγ antibody (1:1000), polyclonal PPARγ antibody (1:1000) at room temperature for 1 h. Then, bands were incubated with specific horse radish peroxidase (HRP)-conjugated secondary antibodies (1:100,000) at room temperature for 1 h and visualized by enhanced chemiluminescence (ECL) substrate with UVP AutoChemi Image system (UVP Inc., Upland, CA, USA). Protein loading was evaluated by anti-actin antibody (1:5000). 

### 4.12. Statistics

Data are expressed as means ± standard deviation. Analysis of variance by Duncan’s test and Pearson’s product-moment correlation coefficient test were determined using SPSS version 10.0 software (SPSS Institute, Inc., Chicago, IL, USA). Differences with *p* < 0.05 were considered statistically significant.

## 5. Conclusions

Combining each of the above test results, in the animal testing, RMD cultured with DOW demonstrated better effects in reducing weight gain, body-fat pads, and the cross-sectional area of adipocytes because, according to the *in vitro* test results, DOW-RMD possessed better ability to inhibit differentiation and LPL activity. Furthermore, DOW-RMD demonstrated better inhibitory effect on differentiation because DOW enhances the rate of monascin and ankaflavin production in RMD, which subsequently increases the ability of RMD to inhibit PPARγ and C/EBPα protein expressions. Moreover, monascin, ankaflavin, and DOW were able to inhibit LPL activity. Thus, DOW-RMD possessed both DOW and relatively high levels of monascin and ankaflavin, all of which facilitated lipogenesis inhibition and consequently prevented the accumulation of lipid droplets. In addition, SW was able to provide SW-RMD with similar anti-obesity effects as that of DOW-RMD, but they resulted from a different mechanism of regulation of differentiation and lipogenesis because of different ion composition. In this study, we simultaneously confirmed that the anti-obesity abilities of DOW-RMD in inhibiting PPARγ and C/EBPα expression in differentiation and lipoprotein lipase activity in lipogenesis were contributed to by the DOW-increased monascin and ankaflavin levels and the ions of DOW, respectively. 

## References

[1] Marques B.G., Hausman D.B., Martin R.J. (1998). Association of fat cell size and paracrine growth factors in development of hyperplastic obesity. Am. J. Physiol..

[2] Kirkland J.L., Hollenberg C.H., Kindler S., Gillon W.S. (1994). Effects of age and anatomic site on preadipocyte number in rat fat depots. J. Gerontol..

[3] Chumlea W.C., Roche A.F., Siervogel R.M., Knittle J.L., Webb P. (1981). Adipocytes and adiposity in adults. Am. J. Clin. Nutr..

[4] Mastinu A., Pira M., Pinna G.A., Pisu C., Casu M.A., Reali R., Marcello S., Murineddu G., Lazzari P. (2013). NESS06SM reduces body weight with an improved profile relative to SR141716A. Pharmacol. Res..

[5] Jou P.C., Ho B.Y., Hsu Y.W., Pan T.M. (2010). The effect of *Monascus* secondary polyketide metabolites, monascin and ankaflavin, on adipogenesis and lipolysis activity in 3T3-L1. J. Agric. Food Chem..

[6] Lee C.L., Wen J.Y., Hsu Y.W., Pan T.M. (2013). Monascus-Fermented yellow pigments monascin and ankaflavin showed antiobesity effect via the suppression of differentiation and lipogenesis in obese rats fed a high-fat diet. J. Agric. Food Chem..

[7] Othmer D.F., Roels O.A. (1973). Power, fresh water, and food from cold, deep sea water. Science.

[8] Fujita D. (2002). Deep ocean water. Shokuhin Eiseigaku Zasshi.

[9] Kimata H., Tai H., Nakagawa K., Yokoyama Y., Nakajima H., Ikegami Y. (2002). Improvement of skin symptoms and mineral imbalance by drinking deep sea water in patients with atopic eczema/dermatitis syndrome (AEDS). Acta Medica (Hradec Kralove).

[10] Kuwayama H., Nagasaki A. (2008). Desalted deep sea water increases transformation and homologous recombination efficiencies in *Dictyostelium discoideum*. J. Mol. Microbiol. Biotechnol..

[11] Hataguchi Y., Tai H., Nakajima H., Kimata H. (2005). Drinking deep-sea water restores mineral imbalance in atopic eczema/dermatitis syndrome. Eur. J. Clin. Nutr..

[12] Hwang H.S., Kim H.A., Lee S.H., Yun J.W. (2009). Anti-Obesity and antidiabetic effects of deep sea water on ob/ob mice. Mar. Biotechnol..

[13] Katsuda S., Yasukawa T., Nakagawa K., Miyake M., Yamasaki M., Katahira K., Mohri M., Shimizu T., Hazama A. (2008). Deep-sea water improves cardiovascular hemodynamics in Kurosawa and Kusanagi-Hypercholesterolemic (KHC) rabbits. Biol. Pharm. Bull..

[14] Lee C.L., Kung Y.H., Wang J.J., Lung T.Y., Pan T.M. (2011). Enhanced hypolipidemic effect and safety of red mold dioscorea cultured in deep ocean water. J. Agric. Food Chem..

[15] Vandenberghe K., Goris M., van Hecke P., van Leemputte M., Vangerven L., Hespel P. (1997). Long-Term creatine intake is beneficial to muscle performance during resistance training. J. Appl. Physiol..

[16] Hallsworth J.E., Nomura Y., Iwahara M. (1998). Ethanol-Induced water stress and fungal growth. J. Ferment. Bioeng..

[17] Giorni P., Magan N., Pietri A., Battilani P. (2011). Growth and aflatoxin production of an Italian strain of *Aspergillus flavus*: Influence of ecological factors and nutritional substrates. World Mycot. J..

[18] Duda V.I., Danilevich V.N., Suzina N.F., Shorokhova A.P., Dmitriev V.V., Mokhova O.N., Akimov V.N. (2004). Changes in the fine structure of microbial cells induced by chaotropic salts. Mikrobiologiia.

[19] Cray J.A., Russell J.T., Timson D.J., Singhal R.S., Hallsworth J.E. (2013). A universal measure of chaotropicity and kosmotropicity. Environ Microbiol.

[20] Hallsworth J.E., Yakimov M.M., Golyshin P.N., Gillion J.L., D'Auria G., de Lima Alves F., La Cono V., Genovese M., McKew B.A., Hayes S.L., Harris G., Giuliano L., Timmis K.N., McGenity T.J. (2007). Limits of life in MgCl_2_-containing environments: Chaotropicity defines the window. Environ. Microbiol..

[21] Lasram S., Oueslati S., Valero A., Marin S., Ghorbel A., Sanchis V. (2010). Water activity and temperature effects on fungal growth and ochratoxin A production by ochratoxigenic *Aspergillus carbonarius* isolated from Tunisian grapes. J. Food Sci..

[22] Cray J.A., Bell A.N., Bhaganna P., Mswaka A.Y., Timson D.J., Hallsworth J.E. (2013). he biology of habitat dominance; Can microbes behave as weeds?. Microb. Biotechnol..

[23] Sepcic K., Zalar P., Gunde-Cimerman N. (2011). Low water activity induces the production of bioactive metabolites in halophilic and halotolerant fungi. Mar. Drugs.

[24] Chen W.P., Ho B.Y., Lee C.L., Lee C.H., Pan T.M. (2008). Red mold rice prevents the development of obesity, dyslipidemia and hyperinsulinemia induced by high-fat diet. Int. J. Obes. (Lond).

[25] Hwang H.S., Kim S.H., Yoo Y.G., Chu Y.S., Shon Y.H., Nam K.S., Yun J.W. (2009). Inhibitory effect of deep-sea water on differentiation of 3T3-L1 adipocytes. Mar. Biotechnol..

[26] Kota B.P., Huang T.H., Roufogalis B.D. (2005). An overview on biological mechanisms of PPARs. Pharmacol. Res..

[27] Rangwala S.M., Lazar M.A. (2004). Peroxisome proliferator-activated receptor gamma in diabetes and metabolism. Trends Pharmacol. Sci..

[28] Yoke Yin C., So Ha T., Abdul Kadir K. (2010). Effects of glycyrrhizic acid on peroxisome proliferator-activated receptor gamma (PPARgamma), lipoprotein lipase (LPL), serum lipid and HOMA-IR in rats. PPAR Res..

[29] Cohen H., Sherer Y., Shaish A., Shoenfeld Y., Levkovitz H., Bitzur R., Harats D. (2002). Atherogenesis inhibition induced by magnesium-chloride fortification of drinking water. Biol. Trace Elem. Res..

[30] Shahkhalili Y., Murset C., Meirim I., Duruz E., Guinchard S., Cavadini C., Acheson K. (2001). Calcium supplementation of chocolate: effect on cocoa butter digestibility and blood lipids in humans. Am. J. Clin. Nutr..

[31] Lee C.L., Hung H.K., Wang J.J., Pan T.M. (2007). Red mold dioscorea has greater hypolipidemic and antiatherosclerotic effect than traditional red mold rice and unfermented dioscorea in hamsters. J. Agric. Food Chem..

[32] Lee C.L., Tsai T.Y., Wang J.J., Pan T.M. (2006). *In vivo* hypolipidemic effects and safety of low dosage *Monascus* powder in a hamster model of hyperlipidemia. Appl. Microbiol. Biotechnol..

[33] Lee C.L., Hung H.K., Wang J.J., Pan T.M. (2007). Improving the ratio of monacolin K to citrinin production of *Monascus purpureus* NTU 568 under dioscorea medium through the mediation of pH value and ethanol addition. J. Agric. Food Chem..

[34] Chen H.C., Farese R.V. (2002). Determination of adipocyte size by computer image analysis. J. Lipid Res..

[35] Folch J., Lees M., Sloane Stanley G.H. (1957). A simple method for the isolation and purification of total lipides from animal tissues. J. Biol. Chem..

[36] Kusunoki M., Hara T., Tsutsumi K., Nakamura T., Miyata T., Sakakibara F., Sakamoto S., Ogawa H., Nakaya Y., Storlien L.H. (2000). The lipoprotein lipase activator, NO-1886, suppresses fat accumulation and insulin resistance in rats fed a high-fat diet. Diabetologia.

[37] Berger J.J., Barnard R.J. (1999). Effect of diet on fat cell size and hormone-sensitive lipase activity. J. Appl. Physiol..

[38] Bihaqi S.W., Singh A.P., Tiwari M. (2012). Supplementation of Convolvulus pluricaulis attenuates scopolamine-induced increased tau and Amyloid precursor protein (AbetaPP) expression in rat brain. Indian J. Pharmacol..

[39] Lee C.L., Kuo T.F., Wu C.L., Wang J.J., Pan T.M. (2010). Red mold rice promotes neuroprotective sAPPalpha secretion instead of Alzheimer’s risk factors and amyloid beta expression in hyperlipidemic Abeta40-infused rats. J. Agric. Food Chem..

